# Supplementary material for: At-Hook Motif Nuclear Localised Protein 18 as a Novel Modulator of Root System Architecture
Source: Int J Mol Sci. 2020 Mar 10;21(5):1886. doi: 10.3390/ijms21051886 (PMC7084884; doi:10.3390/ijms21051886)
Supplement: Supplementary file 1 [file ijms-21-01886-s001.zip › Figure S1 Primers_FINAL.docx]

Table 1 Summary of vector used for Agrobacterium floral dip transformation.

| line | background | Destination Vector | Entry Vector | PCR construct | Primer | Additional restriction site |
| --- | --- | --- | --- | --- | --- | --- |
| pAHL18::AHL18:mRUBY | Col-0 | pBGWT | TaqmRUBY | pAHL18 2,5kb upstream | AHL18_FWD | KpnI |
|  |  |  |  |  | AHL18_REV | SalI |
| pAHl18::GUS | Col-0 | pMDC162 | pENTR A1 | pAHL18 2,2kb upstream | ATH_FW | KpnI |
|  |  |  |  |  | ATH_REV | SalI |
| pAHL18::mCHerry:mCHerry | Col-0 | pB7M35GW | pDONOR221, pEN-R2mCHERRY-L3, pEN-L1-mCHERRY-L2 | pAHL18 1,8kb upstream | pAHL18_B4_FOR |  |
|  |  |  |  |  | pAHL18_B1_REV |  |
| p35S::AHL18:GFP | RDR6 | pGWB5 | pDONOR221 | AHL18 CDS | AHl18_B1_FWD |  |
|  |  |  |  |  | AHL18_B2_REV |  |

Table 2 Primers used for genotyping of homozygous knockout line ahl18.

| Genotyping Primers |  |
| --- | --- |
| SAIL_346_C06_LP | TATCGTTGGGGATGCTCTTAG |
| SAIL_346_C06_RP | ACGTCATGGAGATAACCAACG |
| SAIL_LBb1.3 | ATTTTGCCGATTTCGGAAC |

Table 3 Specific primers for At-hook area of AHL18 used for transcript quantification via qPCR

| q-PCR primers |  |
| --- | --- |
| AHL18_QRT_FW | AACGCTGGACGACAAAAACG |
| AHL18_QRT_REV | GGTTTGTTCTTGGAACCAGCA |
| EF1aF_3 | GACAGGCGTTCTGGTAAGGA |
| EF1aR_3 | GGTGGGTACTCAGAGAAGGT |

Table 4 Primers used for AHL18 PCR construct from gDNA for further cloning.

| Cloning Primers |  |
| --- | --- |
| AHL18_FWD | CTGGTACCTCTGATGCTAAGTGCCG |
| AHL18_REV | TCGTCGACTCCTCTTCTGATTCGC |
| ATH_FW | CTGGTACCTCTGATGCTAAGTGCCG |
| ATH_REV | TCGTCGACTCCTCTTCTGATTCGC |
| pAHL18_B4_FOR | GGGGACAACTTTGTATAGAAAAGTTGCTCTTGGCCTATCGGTTG |
| pAHL18_B1_REV | GGGGACTGCTTTTTTGTACAAACTTGCTCCTCTTCTGATTCGCTAAAA |
| AHl18_B1_FWD | AAAAAGCAGGCTACATGGATGAGGTATCTCGTTCTCATAC |
| AHL18_B2_REV | GGGGACAACTTTGTATAGAAAAGTTGCTCTTGGCCTATCGGTTG |
