# Supplementary material for: At-Hook Motif Nuclear Localised Protein 18 as a Novel Modulator of Root System Architecture
Source: Int J Mol Sci. 2020 Mar 10;21(5):1886. doi: 10.3390/ijms21051886 (PMC7084884; doi:10.3390/ijms21051886)
Supplement: Supplementary file 1 [file ijms-21-01886-s001.zip › Figure S2 + S3 +S4_FINAL.docx]

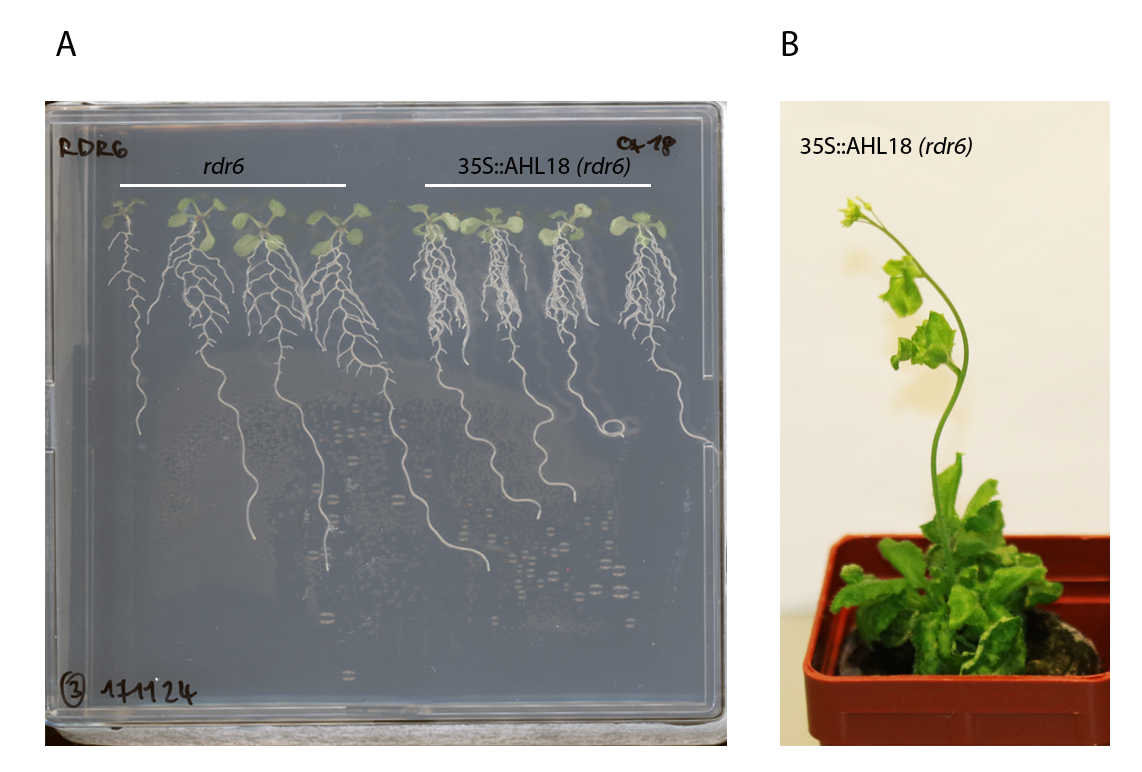


Figure S2 Phenotype of 35S::AHL18 in rdr6 background and rdr6. (A) 11 days old plants grown on MS medium. (B) Four weeks old plant grown peat jiffy.


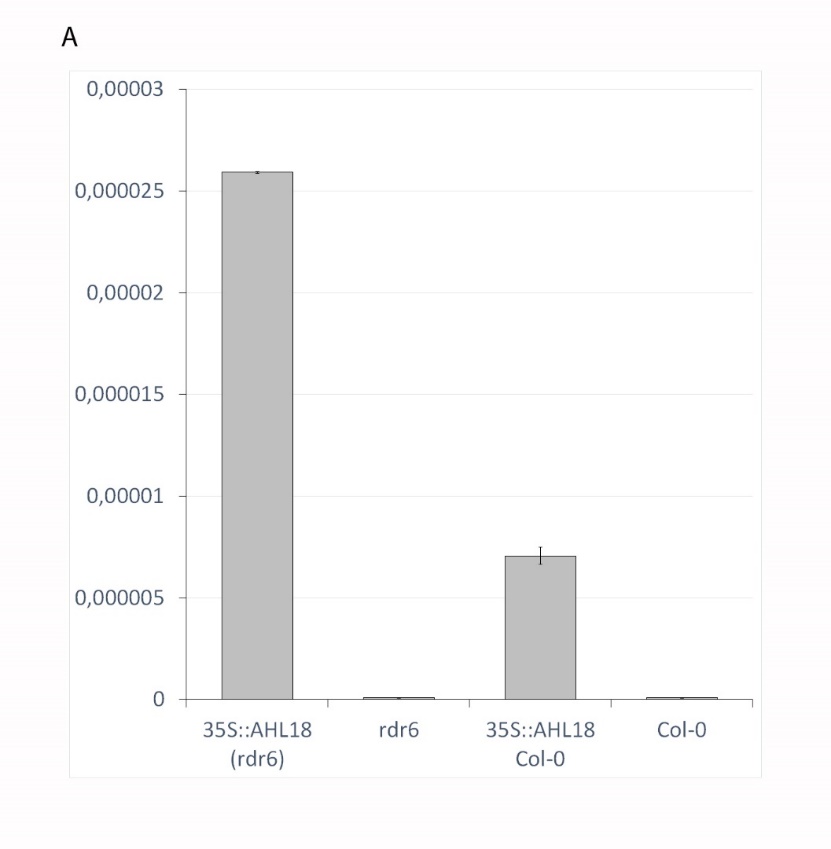


Figure S3 RT-qPCR analysis of *AHL18* transcript levels in AHL18 overexpression lines with different background, Col-0 or rdr6 and respective controls.


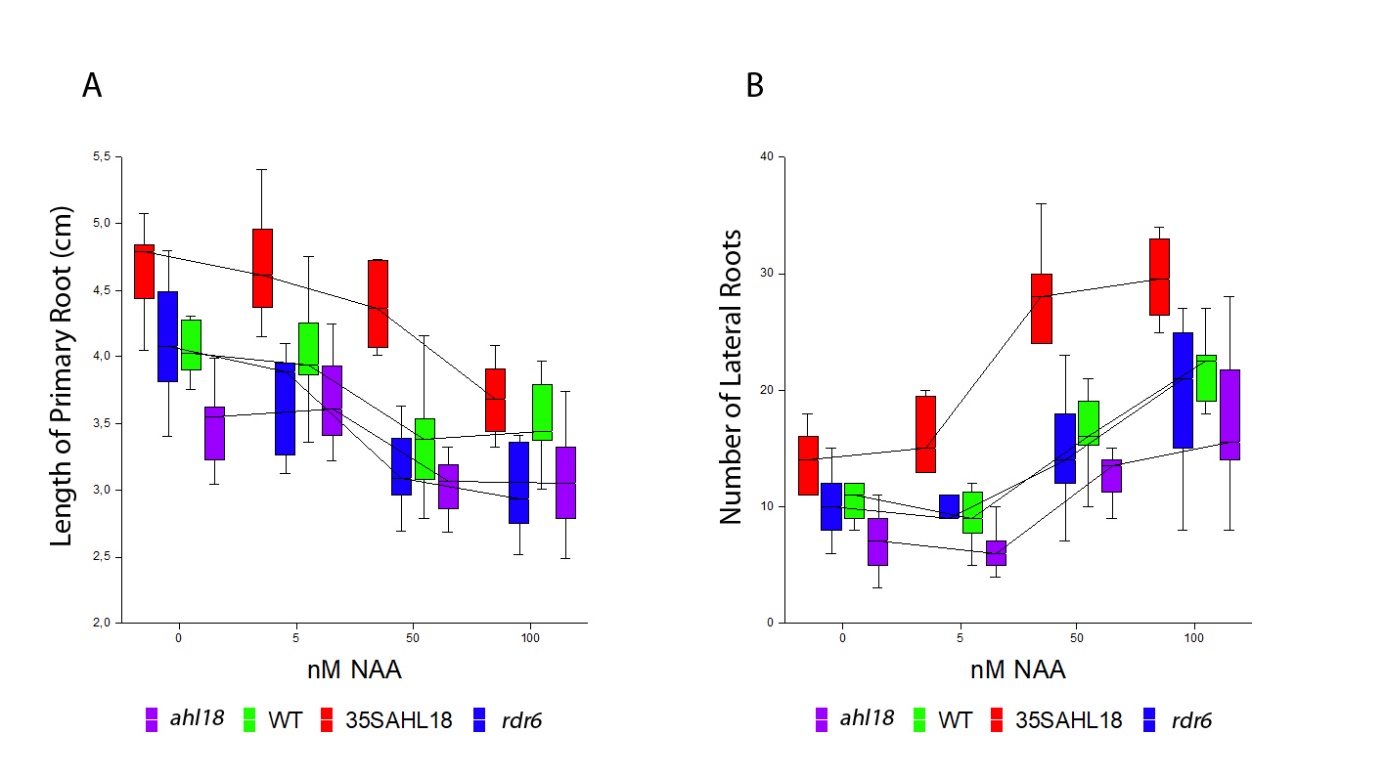


Figure S4 (A) Effect of exogenous auxin on primary root length and (B) number of lateral roots in AHL18 loss-of- and gain-of-function lines. P ≤0,05; ANOVA, n=47 (A,B,C). Data are consistent across two repeated experiments
